# Randomized controlled trials—a critical re-appraisal

**DOI:** 10.1007/s10143-020-01401-4

**Published:** 2020-10-06

**Authors:** Dorothee Mielke, Veit Rohde

**Affiliations:** 1 Department of Neurosurgery, Georg-August-University Göttingen, Robert-Koch-Strasse 40, D-37075 Göttingen, Germany; 2 Osnabrück University of Applied Science, Caprivistraße 1, Osnabrück, 49076 Germany

**Keywords:** Randomized controlled trial, RCT, Fragility index, Evidenced-based medicine, EBM

## Abstract

Randomized controlled trials (RCTs) are considered to represent the gold standard of scientific studies and paved the way for evidence-based medicine (EBM). Besides the initial aim to improve the quality of patient care, EBM is used in the meanwhile for political and economic decision-making and legal issues as well. A review of the literature was performed, followed by a search using links and references of the detected articles. Additionally, homepages for German institutions of public health were screened. Substantial limitations of RCTs and EBM health care could be identified. Based on the selected literature, 80% of the medical treatments have low evidence. RCTs are expensive and are mainly performed by the industry nowadays. A publication bias for positive results exists. Some RCTs are of low external validity. Many studies have a low fragility index. Nonetheless, negative RCTs could be of benefit for the patients. The results of RCTs, gained in a distinct patient population, are partially generalized. RCTs should be analyzed critically before adopting the results to daily clinical routine. It is not really justified to use RCTs and EBM for political and economic decision-making and legal issues as seen today.

## Introduction

The improvement of existing and the development of new therapies are of genuine interest to scientists and clinicians in the different medical disciplines. Knowledge gain is reached by experimental and especially clinical trials of different retrospective and prospective study designs. Retrospective and prospective non-randomized studies are prone to bias, inaccuracy, and missing data and are therefore labeled to be inferior to randomized controlled trials (RCT). The exact design of RCTs was already developed in the 1940s but was largely considered being unethical because for many diseases; at that time, a therapeutic alternative was lacking. In these years, mostly case reports and case series and a few case-control studies were published [1]. Since the 1970s, more than one possibly equally effective therapy for diseases became available in many medical fields. As therapeutic equipoise is considered being the pre-requisite for an RCT by many, a growing acceptance for performing an RCT could be seen over the next decades [9]. The theoretic framework was set by Feinstein in 1967, who published the work *Clinical Judgment* and by Cochrane in 1972, who published the book *Effectiveness and Efficiency: Random reflections on Health Services*, and paved the way to the concept of EBM [5, 7]. Since then, RCTs developed to represent the gold standard of medical research providing the highest classes of evidence (class Ib, if one RCT is available; class Ia, if more than one RCT and a meta-analysis are available) [14].

Hence, RCTs are considered to provide the best available medical knowledge (evidence class I), with the aim to mainly enhance the accuracy of medical decision making and to improve medical therapies [18]. Despite early warnings, a cooptation of RCTs for medico-political, medico-social, medico-legal, and medico-economical decision-making can be currently perceived [2, 8]. The aim of this review is to outline factors that negatively affect the value of RCTs, and to discuss, whether the use of RCTs for decision-making, apart from the mostly specific medical decision-making, is truly being justified.

## Methods

A search of the available literature on RCTs and their limitations was performed using the National Library of Medicine (PubMed), Google, Google Scholar, and Wikipedia. The main search tool was the National Library of Medicine (PubMed). The following MeSH terms were taken: “randomized controlled trial/economics, epidemiology, ethics, legislation, and jurisprudence,” “evidence-based medicine/economics, ethics, history,” and “fragility index.” Only articles in English were included without time restriction. Abstracts were read if the title suggested that the article critically discusses the role of RCTs. The complete paper was read, if the abstract was considered being relevant by the two authors. Additionally, the homepages of the three major German institutions for quality in health care (Ärztliches Zentrum für Qualität in der Medizin [ÄZQ], Arbeitsgemeinschaft medizinischer Fachgesellschaften [AWMF], Bundesministerium für Gesundheit, Institut für Qualität und Wirtschaftlichkeit im Gesundheitsweisen [IQWIG]) were searched for their positioning concerning RCTs and evidence-based medicine (EBM). Because of the very high number of potentially retrievable publications and the blurring difference between more scientific manuscripts and more political statements, expressing the personal opinion of the author, no attempt was made to achieve completeness of the search. Accordingly, the references of the retrieved articles were not used for completing the search.

## Results

### Evidence without RCTs?

Despite the strong focus on RCTs and EBM that could be witnessed in the last 20 years, approximately 50% of all medical therapies are still performed without class I evidence nowadays. Three factors explain this high percentage. (1) Most frequently, the superiority of a therapy/a medical measure is obvious and already proven by studies of lower evidence. Without equipoise, the results of a RCT are predictable, alleviating the reasonableness and possibly the ethical tenability of a study [10]. A neurosurgical example is the role of the electrophysiological monitoring in vestibular schwannoma surgery. Because of the considerable risk of a hearing loss and facial palsy, the integrity of the acoustic and the facial nerve is monitored routinely since the late 1990s, which led to a substantial risk reduction of surgery-associated nerve deficits, as shown by many class II and III studies. (2) Second factor is the low incidence of several, especially pediatric diseases that do not allow the realization of RCTs within an acceptable time frame [15]. (3) Third factor is the technological progress that, in some medical fields, such as cardiology and spine surgery, is faster than the time needed for the completion of a RCT. Consequently, the results are considered to be outdated already in the moment of publication by many [27].

### Economic and ethical aspects

The execution of a RCT is complex and, as a consequence, expensive. The expenses for a phase III prospective, randomized drug trial had been 30 million US dollar at the turn of the millennium [13]; today, the costs are probably substantially higher. Consequently, RCTs initiated and sponsored by the industry outnumber these performed in an academic setting and financed by public funds [3]. This development must be critically assessed concerning several aspects. (1) Not necessarily the scientifically most interesting, but the economically most promising medical questions are being investigated. (2) Drugs or implants that offer no or only slight advantages compared with a competitor are being investigated in RCTs, only because they are being manufactured by another company. Examples are the RCTs including patients with cervical degenerative disc disease, in which different types of total disc replacement (TDR) were compared with anterior discectomy and fusion with almost identical results. (3) Especially in industry-funded RCTs, negative results are less frequently published than positive results [4, 21]. (4) Industry-funded trials, if published, are more frequently cited [17]. (5) Because of the high costs and the strict regulations in first-world countries, an increasing number of industry-sponsored RCTs are performed in second-world countries. Lower health care standards, an underdeveloped understanding of the investigational nature of a trial and a financial compensation for the investigator that is high in comparison with the average local income, might have a substantial influence on the quality of the obtained results. In addition, the participation in a study might be the only access for a patient to any health care, offering the chance of executing studies with an ethically problematic study design [19, 25].

### Methodological aspects (external validity, fragility index)

Before the initiation of a RCT, the number of patients that is required to proof or refute the study hypothesis in a statistically meaningful way has to be calculated. This size of the study population on the one hand and the need to control the costs and to provide results that are still considered to be up-to-date by the time of publication often require a multicenter patient recruitment. Unfortunately, criteria for the selection of participating centers are often not clearly defined. This is especially important for RCTs, in which operative procedures or non-operative management strategies are compared with each other [26]. The individual manual skills and the surgeon’s experience might have a substantial influence on the results. Either in a negative way, if centers with moderate expertise are participating or in a positive way, if highly specialized centers are included [31]. Especially the execution of RCTs in centers with a high expertise (which makes sense in terms of accelerated patient recruitment) leads to the repetitively observable discrepancy between positive study results and the experiences made during clinical routine afterwards. The rigid inclusion criteria of RCTs, mostly performed in the setting of academic hospitals, and the less rigid patient selection during clinical routine in non-academic institutions further increase this discrepancy and reduce the external validity of the specific trial. Sometimes, the positive results of a RCT could not be reproduced in the clinical routine afterwards, representing the lack of external validity of the study [26, 31]. Unfortunately, the external validity of a class I evidence study with a positive result is rarely the content of additional search activities. As an example, we would like to mention a RCT, performed in dedicated neurooncological centers within Europe that compared the overall survival of patients with glioblastoma. The patients either underwent a sole tumor resection or a tumor resection combined with the intraoperative implantation of a local chemotherapeutic agent (carmustine wafer). This study found a significant survival benefit of 2 months in favor of the carmustine wafer group with a comparable complication rate in both groups [30]. After the use in clinical routine, the implantation of carmustine wafers was associated with an increase in the complication rate that hindered the widespread acceptance of this therapy. In most trials, a threshold *p* value of 0.05 is used to determine a statistical significance. The fragility index is the minimum number of events that convert a statistically significant into a statistically insignificant result [29]. Many RCTs have a critically low fragility index. Ridgeon and coworkers evaluated the fragility index of RCTs in critical care medicine [23]. The median fragility index was 2, and 40% of the trials had a fragility index of less than 1. Evaniew et al. evaluated the fragility index of RCTs in spine surgery. They also calculated a median fragility index of 2. In 65% of the included spine studies, the fragility index was less than or equal to the numbers of patients lost to follow-up [6].

### Patient perspective

Primary study endpoints of many RCTs are distinct and easy to evaluate, such as overall survival or progression-free survival in several oncologic trials. Patient-related outcome parameters (PROMs), if assessed at all, are mostly secondary study endpoints [28]. This facilitates data collection, but does not consider the patient perspective sufficiently. Two diametrically opposed effects might be the consequence: A study is positive, but the obtained effect is not noticeable for the patient, or a study is negative, but nonetheless the patient experiences a positive effect [31]. An example for the latter is the GLARIUS trial in patients with a newly diagnosed glioblastoma. The combined use of bevacizumab and irinotecan instead of temozolomide only increased the progression-free survival, but not the overall survival. These findings were classified as a negative result by the German Institute for Quality and Efficiency in Health Care (IQWiG) despite measurable positive effects on the patient’s quality of life [12].

### Generalization and transmission of RCT results

RCTs are designed to answer a distinct medical question in a defined study population. Despite, both negative and positive study results are transferred to patient populations that were not subjects of the trial. An example for such a generalization could be witnessed after the International Subarachnoid Aneurysm Trial (ISAT) [20]. In the ISAT, patients were randomized if the neurosurgeon and neuroradiologist were uncertain about the superior treatment option for the ruptured aneurysm. The key finding of ISAT was a superiority of coiling over clipping in this patient cohort. The precondition of uncertainty for randomization resulted in an underrepresentation of aneurysm locations, in which the neurosurgeon and the neuroradiologist already “knew” the better treatment option: embolization for aneurysms of the posterior circulation and surgery for middle cerebral artery (MCA) aneurysms. In the years after completion of the trial, the scientifically not justified generalization of the study results led to an increasing percentage of MCA aneurysms undergoing coiling. Uncritical acceptance of study results by the health care providers themselves; professional politicians and an influence of the industry are one of the triggers of the aforementioned generalization [22]. Furthermore, positive results, obtained by the use of a technology, that are uncritically transferred to the next generation technology without the scientific proof by a new RCT have to be mentioned. An example is the endovascular treatment of ruptured aneurysms with a WEB device or a stent, which is reasoned with the results of the ISAT study, but which never had been proven to be equal or better than surgery.

## Discussion

The authors acknowledge the important role of RCTs and EBM for improving diagnostics and treatment in medicine, but also believe that certain skepticism should be retained, considering the results of the literature research. Because of the high costs, many RCTs are performed by the industry, which introduces a bias in favor of reporting positive results as witnessed recently for TDR. On the other hand, negative results of industry-sponsored RCTs, which are likewise important from a scientific standpoint, are underreported with a subsequently presumed effect on meta-analyses (which are required for class Ia evidence) towards better results [32]. Less in the field of neurosurgery, but frequently in other medical fields, the high costs seduce the industry to transfer RCTs to the second world, which, apart from the ethical dubiousness, raises the question of the transferability of the results into the first world. Furthermore, we have to be aware that positive results of RTCs are not always reproducible in the “real world” [24]. Finally, several RCTs have a low fragility index, sometimes lower as the lost to follow-up rate.

The intention of the protagonists of RCTs and EBM is considered to be the improvement of medical decision-making, but nowadays RCTs/EBM have gained a substantial political, economic, and legal dimension. In Germany, for example, the IQWiG evaluates the efficacy of new therapies based on RCTs/EBM (https://www.iqwig.de/en/methods/basic-principles.3314.html). That evaluation guides the decision for or against covering the treatment costs by medical insurance companies, which might result in the loss of the patients’ perspective (therapy not paid, but beneficial for the patient and vice versa) [16]. RCTs/EBM are used for the creation of national medical guidelines, which “should support physicians and patients in decision-making for an appropriate treatment of specific health problems” (http://www.awmf.org/leitlinien/awmf-regelwerk/einfuehrung.html), negating the fact that the relevance of RCT results in the non-academic setting, the “real world” is often unclear [26]. Despite not being legally binding, guidelines are increasingly used in medical law suits, with the attempt to judge treatments, not being performed in conformity with guidelines, as incorrect. But, the opposite can be also observed. The lack of class I evidence, despite convincing class II evidence, is being used to exculpate why a standard treatment was not applied [11]. Given the above-mentioned limitations of RCTs, the authors caution against the substantial cooptation of RCTs/EBM for medico-political, medico-social, medico-legal, and medico-economical decision-making.

While RCTs are designed to answer a distinct medical question in a defined study population, we sometimes witness a generalization of the results after the completion of the trial. Typical examples are ISAT and the randomized trial of unruptured brain AVMs (ARUBA) that resulted in an unjustified change of patient management fueled by the interests of neurologists, interventionalists, and neurosurgeons plus the industry (in ISAT). We have to be aware that that generalization of RCTs is scientifically not justified.

## Conclusion

In many instances, RCTs represent the best available scientific evidence. However, RCTs have to be analyzed in detail, and a healthy level of skepticism should be retained, because economic aspects, especially industry funding and methodological flaws, can largely influence the results. The increasing tendency of using RCTs for justification of political, medico-legal, and economic decisions as well as generalizing the results should be seen with caution.
